# Recent Advances in Molybdenum-Based Materials for Lithium-Sulfur Batteries

**DOI:** 10.34133/2021/5130420

**Published:** 2021-03-02

**Authors:** Henghan Dai, Lumin Wang, Yue Zhao, Jialu Xue, Ruicong Zhou, Chenyang Yu, Jianing An, Jinyuan Zhou, Qiang Chen, Gengzhi Sun, Wei Huang

**Affiliations:** ^1^Institute of Advanced Materials (IAM), Nanjing Tech University (NanjingTech), Nanjing 211816, China; ^2^Institute of Photonics Technology, Jinan University, Guangzhou 510632, China; ^3^School of Physical Science and Technology, Lanzhou University, Lanzhou 730000, China; ^4^School of Materials Science and Engineering, Henan Polytechnic University, Jiaozuo 454003, China; ^5^Institute of Flexible Electronics (IFE), Northwestern Polytechnical University (NPU), Xi'an 710072, China

## Abstract

Lithium-sulfur (Li-S) batteries as power supply systems possessing a theoretical energy density of as high as 2600 Wh kg^−1^ are considered promising alternatives toward the currently used lithium-ion batteries (LIBs). However, the insulation characteristic and huge volume change of sulfur, the generation of dissolvable lithium polysulfides (LiPSs) during charge/discharge, and the uncontrollable dendrite formation of Li metal anodes render Li-S batteries serious cycling issues with rapid capacity decay. To address these challenges, extensive efforts are devoted to designing cathode/anode hosts and/or modifying separators by incorporating functional materials with the features of improved conductivity, lithiophilic, physical/chemical capture ability toward LiPSs, and/or efficient catalytic conversion of LiPSs. Among all candidates, molybdenum-based (Mo-based) materials are highly preferred for their tunable crystal structure, adjustable composition, variable valence of Mo centers, and strong interactions with soluble LiPSs. Herein, the latest advances in design and application of Mo-based materials for Li-S batteries are comprehensively reviewed, covering molybdenum oxides, molybdenum dichalcogenides, molybdenum nitrides, molybdenum carbides, molybdenum phosphides, and molybdenum metal. In the end, the existing challenges in this research field are elaborately discussed.

## 1. Introduction

**Figure 1:**
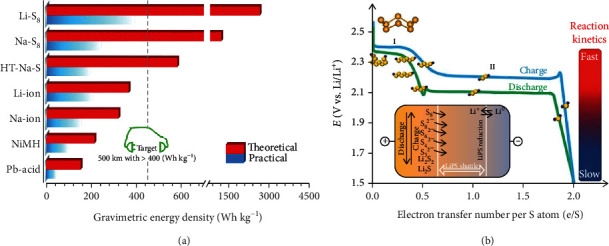
(a) The theoretical and practical gravimetric energy densities of different rechargeable batteries. Reproduced with permission from the Royal Society of Chemistry [17]. (b) The sulfur-based species produced during the charge/discharge process. Inset: the shuttle mechanism of LiPSs. Reproduced with permission from the Royal Society of Chemistry [18].

The rapid development in materials science and technology has boomed the energy storage market, covering widespread applications of smart grids, electric vehicles, portable electronics, etc. [1–8]. Among all currently available battery systems, Li-S rechargeable batteries have drawn great attention because of their cost-effectiveness and extremely high energy density with a theoretical value of 2600 Wh kg^−1^, which is much higher than that of the most advanced LIBs [9–16] and can meet the customers' requirements on electric vehicles with 500 kilometers (corresponding to 500–600 W h kg_sul_^−1^) per charge (Figure 1(a)) [17].

To date, the widely accepted reaction mechanism in Li-S batteries is the multielectron transfer mode (S_8_ + 16Li^+^ + 16e^−^ → 8Li_2_S), involving series reactions of S_8_ → Li_2_S_8_ → Li_2_S_6_/Li_2_S_4_ → Li_2_S_2_/Li_2_S accompanied by a number of intermediates generated during the charge/discharge process (Figure 1(b)) [18]. Since Li_2_S (1.67 g cm^−3^) has a lower density in comparison with sulfur (2.36 g cm^−3^), there involves a volume expansion of ~80% during lithiation, thereby causing electrode degradation/pulverization [17]. Moreover, the insulation features of sulfur and Li_2_S_2_/Li_2_S further hinder the electron transfer and slow down the reaction kinetics [19, 20]. In contrast to Li_2_S_2_ and Li_2_S, the dissolvable lithium polysulfides (LiPSs) diffuse through the porous separator to the negative electrode and react with Li metal forming nondissolvable Li_2_S [21]. Such a “shuttle effect” results in the consumption of sulfur cathodes and the passivation of metal anodes, leading to the increase in internal resistance, the degradation of cycling stability, and the depression of Coulombic efficiency [22–25]. Meanwhile, the Li metal anode also suffers from high chemical reactivity, unstable solid electrolyte interphase (SEI), and dendrite growth during the plating/stripping process, resulting in capacity loss and safety issues [26]. These issues hamper the commercialization of Li-S batteries.

Numerous strategies have been raised to solve the abovementioned problems, such as designing cathode/anode hosts and/or modifying separators [27]. Early tries focused on the incorporation of sulfur into carbonaceous materials, such as graphene foam [28], porous carbon [29], and carbon nanotube network [30], which provide physical constraints on LiPSs. However, the weak intermolecular interaction between nonpolar hydrophobic carbonaceous hosts and polar hydrophobic LiPSs is insufficient to prevent the shuttle of LiPSs. Alternatively, polar substances, e.g., transition metal oxides [31], sulfides [32], and carbides [33], were proposed to enhance the adsorption of LiPSs; nevertheless, the performance improvement remains moderate. Lately, the strategies based on the acceleration of the conversion between LiPSs and Li_2_S_2_/Li_2_S were developed and a number of nanomaterials (e.g., oxides [34], sulfides [35], selenides [36], nitrides [37], carbides, phosphides [38], and metal [39]) have been proven to exhibit catalytic properties. Other viable approaches include the modification of separators to realize the limitation of LiPSs on the cathode side, thereby weakening the shuttle effect, and the protection of Li metal anodes [21]. In general, the overall principle is to incorporate functional materials with the features of improved conductivity, physical/chemical capture ability toward LiPSs, and/or efficient catalytic conversion of LiPSs so as to enhance the device performance.

**Figure 2:**
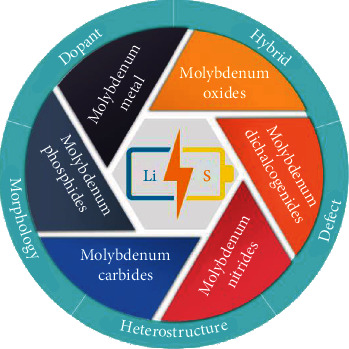
The classification and material design strategies of Mo-based materials for Li-S batteries.

Molybdenum-based (Mo-based) materials are highly preferred due to their tunable crystal structure, adjustable composition, and variable valence of Mo centers, enabling their strong interaction with the soluble LiPSs via a variety of mechanisms for inhibiting the “shuttle effect,” such as polar-polar adsorption, Lewis acid-base interaction, and conversion reaction. Moreover, some Mo-based materials are also reported lithiophilic, which is helpful to suppressing the formation of Li dendrites on anodes and prolonging the cycle life of Li-S batteries [40–42]. Herein, for the first time, we comprehensively review the design and application of Mo-based materials in Li-S batteries (Figure 2), elaborately reveal the interaction between Mo-based materials and LiPSs, and critically discuss the basic mechanism in enhancing adsorption and reaction kinetics. Finally, we summarize the challenges and outline the future prospects of using Mo-based materials in Li-S batteries.

## 2. Molybdenum Oxides

Molybdenum oxides possess a variable valence and adjustable bandgap, which have been widely applied in electronics [43], catalysis [44], energy storage [45], and electrochromic devices [46]. Their crystal and electronic structures can be facilely manipulated through morphology control, defect engineering (e.g., oxygen vacancy and dopants), and composition adjustment. Particularly, in this section, MoO_3_ and MoO_2_ are introduced by discussing their intrinsic properties, modification strategies, and critical roles in Li-S batteries.

### 2.1. MoO_3_

**Figure 3:**
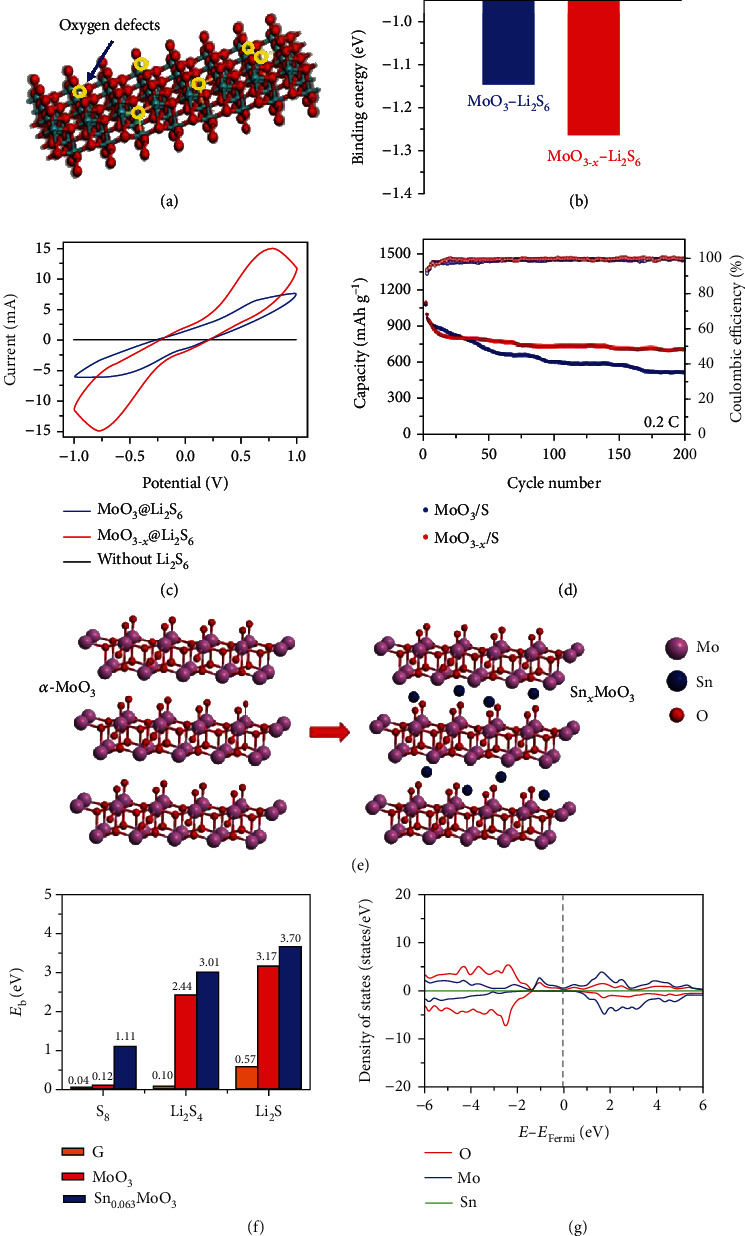
(a) Schematic illustration of the oxygen defects (ODs) on MoO_3−*x*_. (b) The calculated binding energies of Li_2_S_6_ with MoO_3_ and MoO_3−*x*_, respectively. (c) Cyclic voltammetry (CV) curves of symmetric cells based on MoO_3_ and MoO_3−*x*_ electrodes with and without Li_2_S_6_. (d) Cycling performances of MoO_3_/S and MoO_3−*x*_/S cathodes at 0.2 C. (a–d) Reproduced with permission from Wiley-VCH [47]. (e) Schematic illustration showing the intercalation of tin (Sn) atoms into MoO_3_. (f) Calculated binding strength for S_8_, Li_2_S_4_, and Li_2_S on graphene, MoO_3_, and Sn-intercalated MoO_3_ (Sn_0.063_MoO_3_), respectively. (g) Density of states (DOS) for Sn_0.063_MoO_3_. (e–g) Reproduced with permission from Wiley-VCH [48].

MoO_3_ (orthorhombic phase, *α*-MoO_3_) possesses a layered structure composed of thin sheets with a thickness of ~0.7 nm which contains linked and distorted MoO_6_ octahedra [40]. The bandgap of *α*-MoO_3_ is >2.7 eV, and the electrical conductivity was reported to be ~10^−5^ S cm^−1^ [49]. When used as a cathode matrix, the metal-oxygen bond enables *α*-MoO_3_ strong polarity toward efficient LiPS trapping [50]. In this regard, Yi et al. hydrothermally synthesized MoO_3_ nanobelts with a width of ~200 nm and a length of ~1.5 *μ*m, then used them as a cathode matrix for sulfur loading (Figures 3(a)–3(d)) [47]. Based on experimental results and DFT (density functional theory) calculations, they found that oxygen defects (ODs) not only improve the electrical conductivity of MoO_3_ but also obviously enhance the binding strength between MoO_3_ and Li_2_S_6_, effectively anchoring LiPS intermediates during cycling. In addition, both MoO_3_ and MoO_3−*x*_ exhibited catalytic properties toward the reversible conversion of LiPSs tested in symmetric cells using Li_2_S_6_ solution as the electrolyte. Comparably, MoO_3−*x*_ showed higher activity with a large current and distinct redox peaks than MoO_3_, manifesting an enhanced conversion of LiPSs. Compared to MoO_3_/S (1060 mAh g^−1^ at 0.2 C), MoO_3−*x*_/S cathodes delivered a similar capacity of 1076 mAh g^−1^ under 0.2 C with superior cycling stability, retaining 690 mAh g^−1^ after 200 cycles.

In addition to oxygen defects, the intrinsic properties of MoO_3_ can also be adjusted by inserting guest atoms or molecules into their van der Waals interlayer gaps [51]. Following this direction, Yang et al. prepared tin- (Sn-) intercalated MoO_3_ (Sn_0.063_MoO_3_) via the disproportionation of Sn(II) (Figures 3(e)–3(g)) [48]. DFT calculations indicated that the electron transferred from the intercalated Sn atoms to MoO_3_ resulted in the emergence of the spreading states around the Fermi level. This led to enhanced electrical conductivity and binding energies of Li_2_S_4_ and S_8_ to Sn_0.063_MoO_3_, which therefore effectively improved the cathode rate performance and depressed the LiPS shuttle. The as-fabricated Li-S battery delivered an initial capacity of 906 mAh g^−1^ at 1 C with 79.6% retention after 500 cycles.

**Figure 4:**
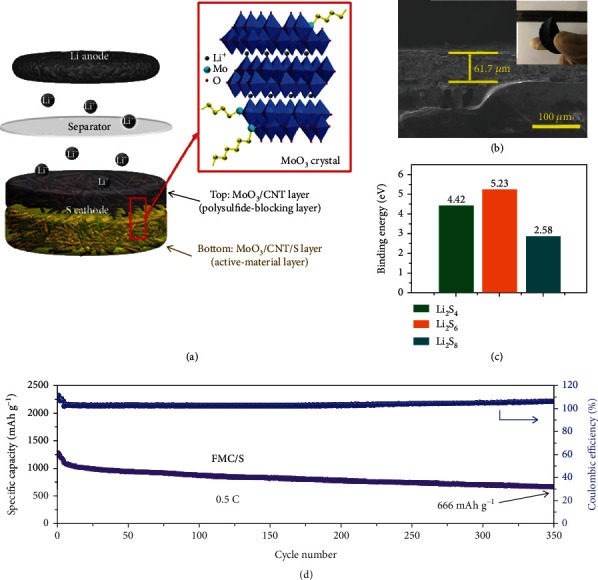
(a) Li-S battery adopting freestanding MoO_3_/CNT/S (FMC/S) as the cathode. (b) Cross-sectional observation of the FMC/S membrane. Inset: bending test of the FMC/S membrane. (c) The calculated binding energies between MoO_3_ and LiPSs (including Li_2_S_4_, Li_2_S_6_, and Li_2_S_8_). (d) Cycling stability of the FMC/S at 0.5 C. (a–d) Reproduced with permission from the American Chemical Society [52].

Hybridizing with carbonaceous materials is another viable way to mitigate the low electrical conductivity of MoO_3_. A typical work was presented by Chen et al., where a freestanding membrane containing a cathode layer of MoO_3_/CNT/S (FMC/S) and a LiPS-blocking layer of intertwined MoO_3_/CNTs was fabricated via a sequential filtration method (Figure 4) [52]. In this manner, the interfacial binding strength between the two layers was improved, and the poor conductivities of sulfur and MoO_3_ were effectively alleviated. Combined with the strong polarity of *α*-MoO_3_, LiPSs were effectively trapped. The device delivered a specific capacity of 1074 mAh g^−1^ at 0.5 C, retaining 666 mAh g^−1^ after 350 cycles.

**Figure 5:**
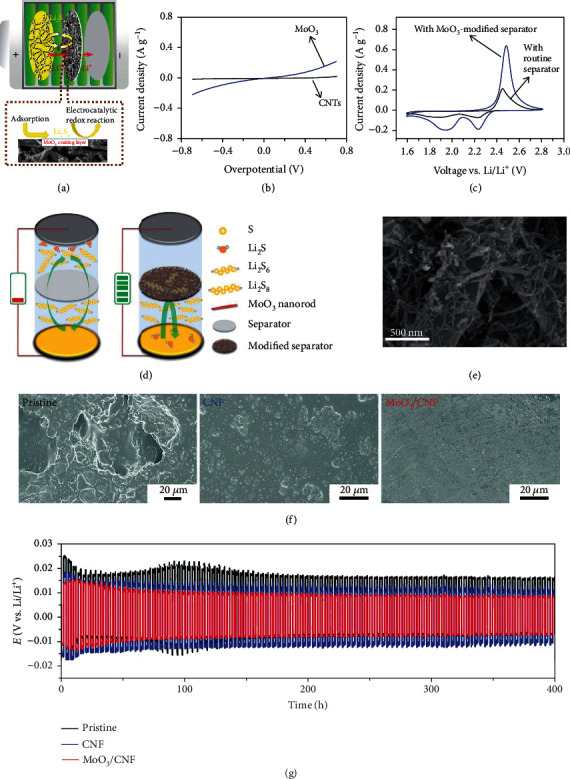
(a) Li-S battery based on the CNT/S cathode together with a separator modified by MoO_3_. (b) Polarization curves of Li-S cells containing Li_2_S_6_ in a symmetric configuration. (c) CV behaviors of the Li-S battery containing the MoO_3_-modified separator in comparison with that based on the routine separator. (a–c) Reproduced with permission from Wiley-VCH [53]. (d) Li-S batteries based on pristine and modified (MoO_3_@CNT) separators. (e) SEM image of the MoO_3_@CNT network. (d, e) Reproduced with permission from the Royal Society of Chemistry [54]. (f) The surface morphology of Li metal anodes with pristine, CNF-modified, and MoO_3_/CNF-modified separators after cycling at 1 C for 500 times. (g) Voltage-time profiles of the symmetric batteries (Li|Li) with pristine, CNF-modified, and MoO_3_/CNF-modified separators (testing conditions: 0.5 mA cm^−2^ and 1 mAh cm^−2^). (f, g) Reproduced with permission from the Royal Society of Chemistry [55].

In addition to designing a cathode matrix, the idea of modifying separators was adopted to relieve the shuttle effect of LiPSs, which is comparably a low-cost strategy [56]. Imtiaz et al. coated MoO_3_-based slurry onto the commercial separator forming a porous network (Figures 5(a)–5(c)) [53]. Such a hybrid separator in Li-S batteries enabled fast ion transportation. Due to the catalytic property of MoO_3_ and the intimate contact between the cathode and the separator, the as-assembled symmetric cell provided increased current density and sharper redox peaks compared to that based on the routine separator and CNT-modified separator. The as-fabricated Li-S battery exhibited a specific capacity of 1377 mAh g^−1^ at 0.5 C with retention of 49.7% after 200 cycles. Following this idea, Kaisar et al. further designed a modified polypropylene separator with lithium-passivated MoO_3_ nanobelts [57]. The as-prepared battery achieved an improved capacity at 0.5 C (717 mAh g^−1^ after 500 cycles), attributable to (i) the strong adsorption of MoO_3_ to LiPSs and (ii) the increased conductivity of MoO_3_ owing to the lithiation (Li*_x_*MoO_3_) [58]. Further enhancement in the adsorption ability of MoO_3_ can be achieved by hybridizing with carbonaceous materials. The interwoven MoO_3_@CNT-modified separator fabricated via the vacuum filtration method by Luo et al. not only provided abundant charge (electrons and ions) transport pathways but also effectively mitigated the LiPS shuttle (Figures 5(d) and 5(e)) [54]. The resultant Li-S cell showed a specific capacity of 1070 mAh g^−1^ at 0.3 C with 61.2% retained at 3 C (655 mAh g^−1^). Moreover, when cycling at 1 C for 400 cycles, the device maintained 53.4% of the initial capacity, corresponding to 641 mAh g^−1^.

Since Li metal has high activity, the presence of LiPSs leads to the generation of insulating Li_2_S_2_/Li_2_S on the Li surface, promoting the formation of Li dendrites and shortening the anode lifespan. Therefore, in addition to the enhancement of cathode performance, the effective inhibition of LiPS shuttling also protects Li metal anodes from the corrosion by LiPSs. In a recent work shown in Figures 5(f) and 5(g), a freestanding MoO_3_/carbon nanofiber (MoO_3_/CNF) membrane was prepared by solvothermally depositing MoO_3_ nanoparticles on the carbonized electrospun PAN nanofibers and used as an interlayer in Li-S batteries [55]. The combination of the polar MoO_3_ and conductive CNF network efficiently facilitates the conversion between Li_2_S_2_/Li_2_S and sulfur species, suppressing LiPS shuttling. The symmetric battery (Li|Li) with the MoO_3_/CNF interlayer exhibited improved cycling stability over 400 h of testing at 0.5 mA cm^−2^ under 1 mAh cm^−2^ and smaller overpotential than the devices made of the CNF interlayer and pristine separator (Figure 5(g)).

### 2.2. MoO_2_

**Figure 6:**
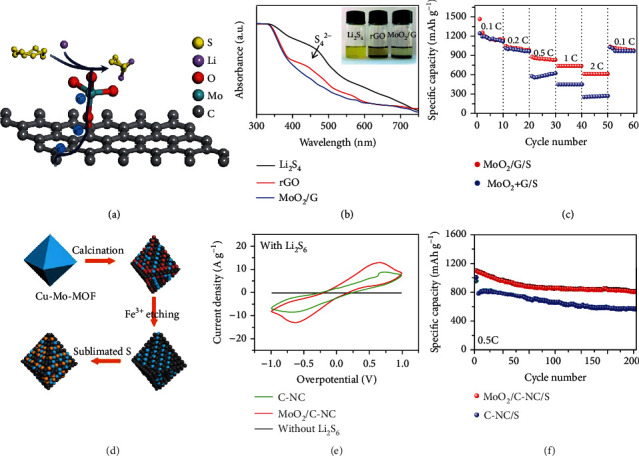
(a) The charge transfer between MoO_2_/G and LiPSs. (b) The adsorption capabilities of MoO_2_/G and graphene to Li_2_S_4_ evidenced by UV-vis spectra. (c) The rate performance of MoO_2_/G/S cathodes compared to that of MoO_2_+G/S. (a–c) Reproduced with permission from the Royal Society of Chemistry [59]. (d) The synthesis of MoO_2_/C-NC/S. (e) CV curves of the symmetrical Li_2_S_6_ cells. (f) The stability of MoO_2_/C-NC/S and C-NC/S cathodes at 0.5 C. (d–f) Reproduced with permission from the American Chemical Society [60].

Different from MoO_3_, monoclinic MoO_2_ has a deformed rutile structure with a tetragonal orientation, where the MoO_6_ octahedra share the opposite edges along the crystallographic *c*-axis [40]. Typically, it displays higher electrical conductivity compared with MoO_3_, due to the small bandgap [61]. Wu et al. grew MoO_2_ hollow spheres on N-doped graphene (MoO_2_/G) via hydrothermal reaction and used them as the sulfur host (Figures 6(a)–6(c)) [59]. In comparison with the physical mixture of MoO_2_ and graphene, MoO_2_/G exhibited enhanced rate performance and stability attributable to the enlarged accessible surface of N-doped graphene, the strong interaction between LiPSs and MoO_2_, and the efficient electron transfer between N-doped graphene and MoO_2_ via the C-O-Mo bond. MoO_2_/G/S delivered specific capacities of 810 mAh g^−1^ at 1 C and maintained 664 mAh g^−1^ after 500 cycles.

Porous structure was proposed to alleviate the volume expansion of sulfur during lithiation and in the meantime restrict the LiPS shuttle. Wang et al. prepared porous frameworks composed of MoO_2_ and carbon (MoO_2_/C-NC) through the carbonization of Cu-Mo-MOF (metal-organic frameworks) followed by FeCl_3_ etching (Figures 6(d)–6(f)) [60]. Sulfur was homogeneously distributed in porous MoO_2_/C-NC. In comparison with the bare carbonaceous host, MoO_2_/C-NC exhibited high electrical conductivity and strong interactions to LiPSs via Li-O and Mo-S bonds. As a result, the MoO_2_/C-NC-based symmetrical cells presented improved reaction kinetics with higher current density and lowered overpotential with minimized potential separation between redox peaks, confirming that MoO_2_ accelerated the electrochemical reactions of LiPSs. At 0.5 C, the MoO_2_/C-NC/S electrode delivered 801 mAh g^−1^ after 200 cycles, corresponding to 73.4% retention of the initial capacity. Following this direction, Razaq et al. prepared the MoO_2_/rGO host by annealing Mo-based MOF (Mo-MOF) wrapped with graphene oxide (GO) in Ar [62]. The obtained MoO_2_ microrods featured crispy rice-like mesoporous structure and exhibited high electron and Li^+^ conductivity, good confinement for LiPSs, and catalytic conversion of LiPSs to thiosulfates (polythionates). Consequently, the MoO_2_/rGO/S cathode showed good charge/discharge stability at 0.5 C for 500 cycles with a capacity of 1027 mAh g^−1^, corresponding to 90.0% retention of the initial value.

**Figure 7:**
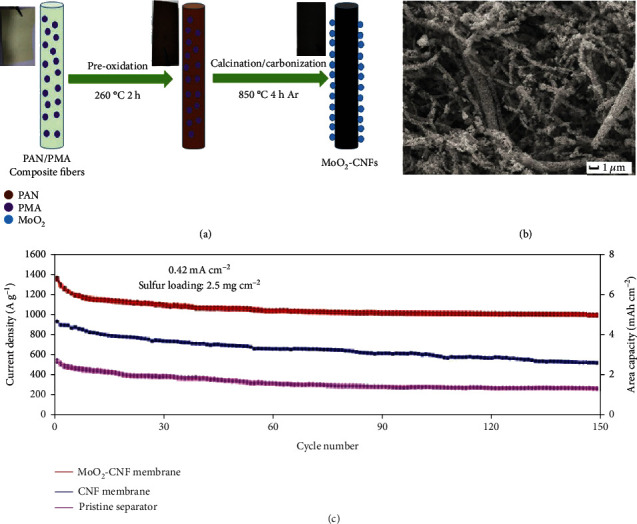
(a) Formation process of MoO_2_-CNF. (b) SEM image of MoO_2_-CNF. (c) Cycling stabilities of the Li-S battery without the interlayer and the devices with interlayers of CNF and MoO_2_-CNF, respectively. (a–c) Reproduced with permission from Wiley-VCH [63].

The interlayer based on the combination of MoO_2_ and carbon materials was inserted between the sulfur cathode and the separator [64]. Zhuang et al. incorporated MoO_2_ nanoparticles into carbon nanofibers by carbonizing the electrospun membrane that consisted of PAN fibers and phosphomolybdic acid (PMA: H_3_PO_4_·12MoO_3_) (Figure 7) [63]. The obtained MoO_2_-CNF interlayer can effectively trap LiPSs and improve the reversibility of sulfur reaction during charge/discharge. The as-fabricated battery with the MoO_2_-CNF interlayer showed capacity retention of 73.0% at 0.4 mA cm^−2^ after 150 cycles, corresponding to 1006 mAh g^−1^.

Molybdenum oxides have strong polarity with Lewis acid sites (Mo) and Lewis base sites (O) for capturing LiPSs and the subsequent catalytic conversion. However, they typically have low conductivity and small specific surface areas [23], which are not conducive to the electron transfer and LiPS adsorption/conversion during cycling. Therefore, in order to improve their performance in Li-S batteries, defect engineering (e.g., oxygen vacancies), intercalation (e.g., Sn), hybridization with conductive filaments (e.g., CNT and rGO), and structure design (e.g., hollow cages and porous frameworks) are highly necessitated.

## 3. Molybdenum Dichalcogenides

During the past decade, the 2D molybdenum dichalcogenides (e.g., MoS_2_ and MoSe_2_) have drawn great attention due to their unique features of tunable compositions, crystal structures, valence states, and morphologies, endowing them with high electrochemical activities and potential applications in energy storage [35, 36, 65–68]. In this section, recent advances in these two kinds of molybdenum dichalcogenides are discussed.

### 3.1. MoS_2_

**Figure 8:**
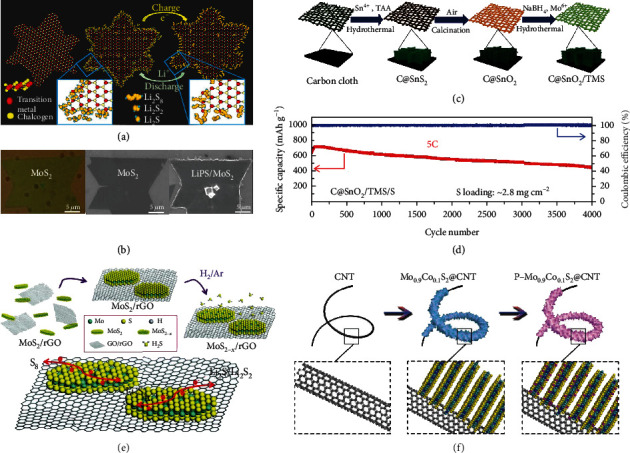
(a) Schematic illustration for the confined deposition and conversion of LiPSs at the preferential catalytic sites of transition metal dichalcogenide (TMD) nanosheets. (b) Optical and SEM images of MoS_2_ flakes and LiPS-deposited MoS_2_ flakes at the discharged state. (a, b) Reproduced with permission from the American Chemical Society [69]. (c) The synthesis of the hierarchical C@SnO_2_/TMS nanoarray on carbon cloth. (d) Long-term cycling stability test of the C@SnO_2_/TMS/S cathode at 5 C. (c, d) Reproduced with permission from the American Chemical Society [70]. (e) The synthesis of MoS_2−*x*_/rGO and the conversion of Li_2_S*_x_*. Reproduced with permission from the Royal Society of Chemistry [71]. (f) The synthesis of cobalt (Co) and phosphorus (P) codoped MoS_2_ (P-Mo_0.9_Co_0.1_S_2_) on CNT. Reproduced with permission from Wiley-VCH [72].

Single-layer molybdenum disulfide (MoS_2_) is composed of Mo (+4) and S (−2) atoms arranged into a sandwiched structure via covalent bonds of S-Mo-S, whereas MoS_2_ nanosheets are held together through relatively weak interaction of van der Waals forces [73]. Due to the unsaturated bonding at the defect sites (e.g., edge and vacancies), MoS_2_ facilitates the catalytic conversion of LiPSs [74–76]. Babu et al. synthesized MoS_2_ nanoflakes by chemical vapor deposition (CVD) and cycled them against lithium foil in a cell containing LiNO_3_, LiTFSI, and Li_2_S_4_ as the catholyte [69]. The experimental observation showed the unsaturated sulfur sites on the edge of MoS_2_ facilitated the adsorption and subsequent catalytic conversion of LiPSs to Li_2_S_2_/Li_2_S (Figures 8(a) and 8(b)).

Recently, the combination of metal sulfides and oxides has been confirmed to offer improved adsorption ability toward LiPSs. In a typical work, Wang et al. grew C@SnO_2_/1T-MoS_2_ (C@SnO_2_/TMS) arrays with hierarchical architectures on carbon cloth via hydrothermal reaction and used them as the host for sulfur [70]. In this hierarchical structure, SnO_2_ nanosheets that consisted of small nanoparticles were coated by 1T-MoS_2_ (Figures 8(c) and 8(d)). On the one hand, the porous structure effectively suppressed the volume change of sulfur and in the meantime allowed fast transportation of Li^+^ [77]. On the other hand, SnO_2_ provided stronger adsorption to Li_2_S_4_ compared to MoS_2_ (0.46 eV) with a binding energy of 2.64 eV according to DFT calculations, while 1T-MoS_2_ nanosheets with high conductivity and abundant edges accelerated the redox kinetics of LiPSs effectively. The resultant C@SnO_2_/TMS/S cathode delivered 710 mAh g^−1^ at 5 C with 63.0% retained after 4000 cycles.

The catalytic activity of MoS_2_ can be further tuned via defect engineering. For instance, Lin et al. introduced sulfur vacancies in MoS_2_ and evaluated its effect on the catalytic conversion of LiPSs (Figure 8(e)) [71]. In a typical synthesis, MoS_2_ nanoflakes were prepared by liquid-phase exfoliation and mixed with GO by filtration. Such composites were thermally treated in hydrogen at 600°C for 6 h. The amount of sulfur deficiencies was varied by changing annealing duration and temperature. The catalytic behavior of MoS_2−*x*_ on the conversion of LiPSs was revealed in symmetric cells with 0.2 M Li_2_S_6_ as the electrolyte. The results indicated that the sulfur deficiencies in MoS_2−*x*_ rendered MoS_2−*x*_/rGO more active sites and facilitated the redox conversion of sulfur to LiPSs. The sulfur cathode with 4.0 wt% of MoS_2−*x*_/rGO showed specific capacities of 1159 mAh g^−1^ and 827 mAh g^−1^ at 0.5 C and 8 C, respectively. Liu et al. incorporated defect-rich MoS_2_ into porous graphene aerogel (denoted as GA-DR-MoS_2_) and further confirmed that abundant defects assisted efficient adsorption and catalytic reactions of LiPSs during electrochemical cycling [78]. The resultant GA-DR-MoS_2_-based cathode containing 70.0 wt% of sulfur presented a discharging capacity of 581 mAh g^−1^ under 5 C.

In another work, Lin et al. decorated moss-like Mo_0.9_Co_0.1_S_2_ nanosheets on the CNT surface by the hydrothermal method forming a core-shell tubular structure followed by phosphorus doping (P doping) at elevated temperature using red phosphorus as the precursor (Figure 8(f)) [72]. The experimental results demonstrated that MoS_2_ nanotubes codoped by Co and P atoms improved the catalytic conversion of LiPSs in both directions (the sulfur reduction and the sulfur evolution). Particularly, Co doping induced the formation of 1T-MoS_2_, guaranteeing low electron transport resistance, while P dopants provided an electron-rich environment in the Mo_0.9_Co_0.1_S_2_, which was conducive to the scission of the S-S bonds. Consequently, the P-Mo_0.9_Co_0.1_S_2_/S showed 1187 mAh g^−1^ at 0.5 C after 150 cycles, corresponding to 89.0% retention of the initial capacity.

**Figure 9:**
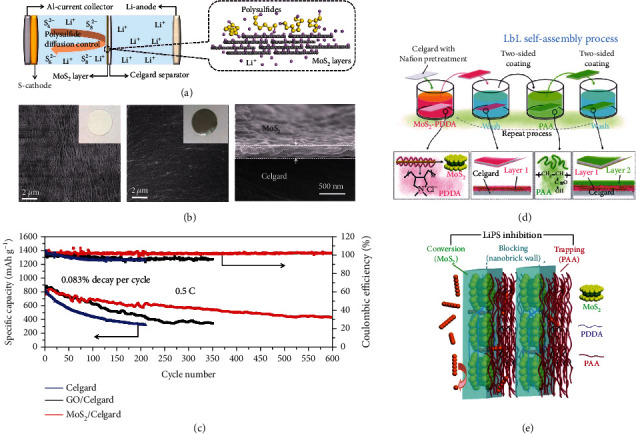
(a) Li-S battery based on the MoS_2_/Celgard separator. (b) SEM images of the Celgard surface (inset: the photograph of pristine Celgard), MoS_2_/Celgard surface (inset: the photograph of MoS_2_-coated Celgard), and cross-section of MoS_2_ layers. (c) Long-term cycling tests of Li-S cells constructed by MoS_2_/Celgard, GO/Celgard, and Celgard separators. (a–c) Reproduced with permission from Wiley-VCH [79]. (d) The preparation of the MoS_2_-PAA-modified separator assembled in a layer-by-layer manner. (e) The trapping and conversion of LiPSs on the MoS_2_-PAA-modified separator. (d, e) Reproduced with permission from Wiley-VCH [80].

Similar to molybdenum oxides, MoS_2_ was also used to modify the separator as a barrier to alleviate the LiPS shuttle effect. As a typical example, Ghazi et al. exfoliated MoS_2_ nanosheets via Li^+^ intercalation and filtrated them on commercial Celgard separators (MoS_2_/Celgard) for Li-S batteries (Figures 9(a)–9(c)) [79]. Electrochemical impedance spectroscopy (EIS) disclosed that MoS_2_/Celgard showed rapid Li^+^ diffusion with similar conductivity (2.0 × 10^−1^ mS cm^−1^) to bare Celgard (3.3 × 10^−1^ mS cm^−1^) but much higher than GO/Celgard (3.1 × 10^−2^ mS cm^−1^). The reason was assigned to the high Li^+^ density on the MoS_2_ surfaces generated during exfoliation. In addition, the MoS_2_/Celgard separator also effectively blocked LiPSs due to the presence of MoS_2_. The battery with the MoS_2_/Celgard separator exhibited 808 mAh g^−1^ at 0.5 C initially and retained 401 mAh g^−1^ after 600 cycles. Zheng et al. modified the Celgard separator by edge-rich MoS_2_/C hollow microspheres (Edg-MoS_2_/C HMs) by hydrothermal reaction [81]. The Edg-MoS_2_/C HMs enabled the efficient conversion of LiPSs and provided abundant sites for Li_2_S absorption. The as-fabricated cells with sulfur loading of 1.7 mg cm^−2^ and 6.1 mg cm^−2^ delivered capacities of 896 mAh g^−1^ and 554 mAh g^−1^ at 0.5 C, respectively. Wu et al. designed a separator with dual functionality via a layer-by-layer self-assembly strategy (Figures 9(d) and 9(e)) [80]. The positively charged MoS_2_-poly(diallyl dimethyl ammonium chloride) (PDDA) (denoted as M-P) hybrid and the negatively charged poly(acrylic acid) (PAA) were alternatively deposited on the commercial separator (denoted as M-P/P) forming a “nanobrick wall” structure. The PAA mortars selectively impeded the travel of large LiPSs (1–2 nm) compared to Li^+^ because of their strong binding energies toward Li_2_S_2_, Li_2_S_4_, and Li_2_S_6_, while well-orientated MoS_2_ nanosheet bricks catalyzed the conversion of LiPSs to the insoluble thiosulfate and polythionate complex, which further anchored LiPSs from solution and ultimately converted to Li_2_S_2_/Li_2_S. As a result, the separator with 0.1 mg cm^−2^ of M-P/P coating endowed the Li-S battery 423 mAh g^−1^ after 2000 cycles at 1 C, corresponding to 42.0% retention of the initial capacity.

**Figure 10:**
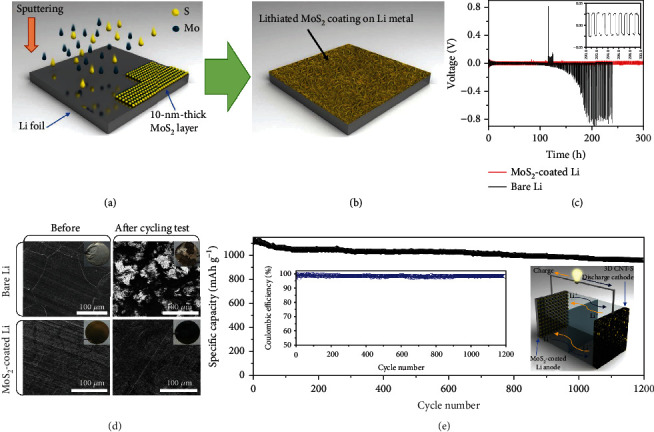
(a) The sputtering and (b) subsequent lithiation of MoS_2_ for the modification of the Li anode. (c) Voltage-time profiles of symmetric cells using bare Li and MoS_2_-modified Li as anodes tested under 10.0 mA cm^−2^. Inset is the magnified voltage-time profile for the MoS_2_-modified Li anode. (d) The SEM and photo (inset) images of bare Li and MoS_2_-modified Li during cycling at 10.0 mA cm^−2^. (e) The stability of the Li-S cell composed of the MoS_2_-modified Li anode and CNT-S cathode cycling over 1200 times at 0.5 C. Insets are the Coulombic efficiency (left) and schematic kinetics of the Li-S battery. (a–e) Reproduced with permission from Nature [82].

In 2018, Cha and coworkers made the first attempt to protect Li metal anodes by sputtering a thin layer of 2H MoS_2_ with a thickness of ~10 nm (Figure 10(a)) [82]. The subsequent lithiation transformed the crystal phase of MoS_2_ from semiconducting 2H to metallic 1T (Figure 10(b)), thereby lowering the interfacial impedance (between the Li metal and the electrolyte). Moreover, 1T-MoS_2_ has a small Li migration energy barrier of 0.155 eV, which is beneficial for the rapid diffusion of Li^+^ to Li metal and the homogeneous Li deposition. The modification of lithiated MoS_2_ led to low-voltage polarization of ~52 mV at 10.0 mA cm^−2^, a threefold improvement in the cycle life compared to bare Li metal, and effective suppression of Li dendrites (Figures 10(c) and 10(d)). The Li-S batteries made of the CNT-S cathode and MoS_2_-modified Li anode exhibited a high capacity of 1105 mAh g^−1^ with excellent retention of 84.0% over 1200 cycles at 0.5 C (Figure 10(e)).

### 3.2. MoSe_2_

**Figure 11:**
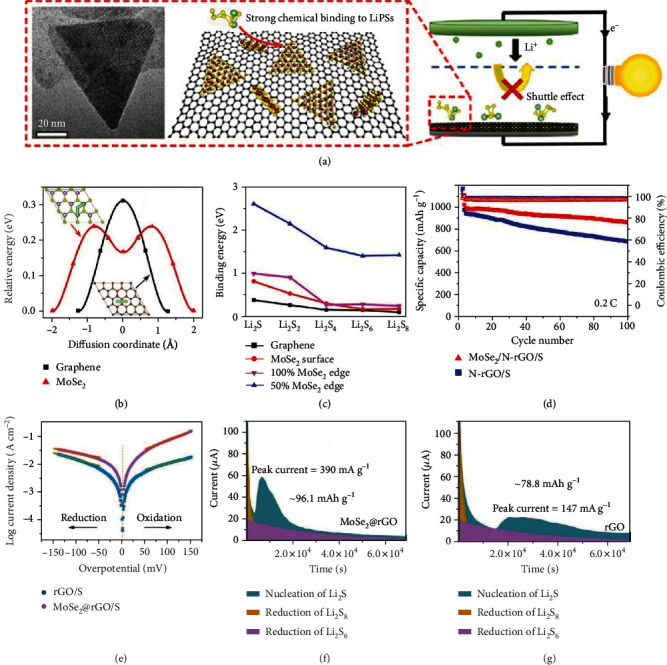
(a) Schematic of the MoSe_2_ on nitrogen-doped reduced GO (MoSe_2_/N-rGO) and its strong chemical binding to LiPSs. (b) Energy profile of Li atom diffusion on graphene and MoSe_2_ surfaces. (c) Binding energy of Li_2_S*_n_* on the graphene surface, MoSe_2_ surface, 100% MoSe_2_ edge, and 50% MoSe_2_ edge. (d) Cycling stability of MoSe_2_/N-rGO/S and N-rGO/S. (a–d) Reproduced with permission from the American Chemical Society [83]. (e) The Tafel plots of the MoSe_2_@rGO/S and rGO/S cathodes. The discharge behavior of Li_2_S_8_ on (f) MoSe_2_@rGO and (g) rGO at 2.05 V. (e–g) Reproduced with permission from Wiley-VCH [84].

Molybdenum diselenides (MoSe_2_) have been applied in LIBs as anode materials since the 1970s because of their high capacity and long cycle life [36, 85, 86]. Similar to MoS_2_, MoSe_2_ also exhibit preferential adsorption of LiPSs at the edge sites due to the unsaturated bonding of Se [87]. Wong et al. synthesized the MoSe_2_/N-rGO hybrid as the sulfur host for Li-S batteries. The triangular-shaped MoSe_2_ with a lateral size of 10–60 nm were loaded on N-doped graphene by the CVD method (Figure 11(a)) [83]. Theoretical calculation (Figures 11(b) and 11(c)) indicated that MoSe_2_ exhibited a lower Li diffusion energy barrier of 0.2374 eV compared with graphene (0.3104 eV). The obtained MoSe_2_/N-rGO/S electrode delivered a capacity of 887 mAh g^−1^ after charged/discharged at 0.2 C for 100 cycles (Figure 11(d)), corresponding to 86.3% retention.

Tian et al. decorated MoSe_2_ nanoflakes on rGO using hydrothermal reaction and employed linear sweep voltammetry (LSV) at the full discharge state and potentiostatic method to study the catalytic properties of MoSe_2_ on the LiPS conversion in Li-S batteries (Figures 11(e)–11(g)) [84]. The experimental results showed that the presence of MoSe_2_ facilitated the full conversion of LiPSs and nucleation of Li_2_S. Consequently, the MoSe_2_@rGO/S cathode retained 941 mAh g^−1^ (78.4% of the initial capacity) after charged/discharged for 200 cycles at 0.5 C.

Molybdenum dichalcogenides show site-selective catalytic performance and phase-dependent conductivity. Typically, defect sites (e.g., edge and vacancies) exhibit higher catalytic activity than basal planes, while the 1T phase has a lower energy barrier for both electron transport (facilitating catalytic conversion) and Li^+^ migration (suppressing Li dendrites) than 2H. However, the adsorption capability of molybdenum dichalcogenides is moderate compared to that of molybdenum oxides. Moreover, the synthesis of 1T phase molybdenum dichalcogenides usually requires complex procedures involving ion intercalation, heteroatom doping, and utilization of explosive reagents.

## 4. Molybdenum Nitrides

**Figure 12:**
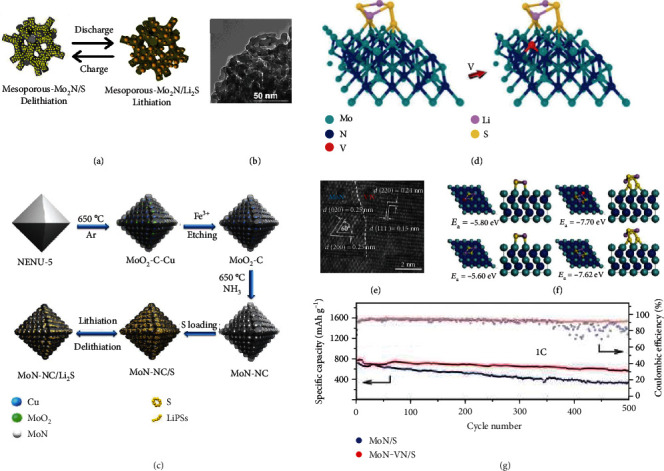
(a) The charge/discharge process of the mesoporous Mo_2_N/S cathode. (b) TEM image of mesoporous Mo_2_N. (a, b) Reproduced with permission from Elsevier [92]. (c) Synthesis of MoN-NC. Reproduced with permission from the Royal Society of Chemistry [93]. (d) The model of the 2D MoN-VN heterostructure. (e) The scanning transmission electron microscopy (STEM) image of the MoN-VN. (f) Optimized configurations and binding energies of V-MoN-Li_2_S_2_, V-MoN-Li_2_S_4_, MoN-Li_2_S_2_, and MoN-Li_2_S_4_, respectively. (g) Cycling performances and CE of MoN/S and MoN-VN/S cathodes. (d–g) Reproduced with permission from Wiley-VCH [94].

Transition metal nitrides are widely adopted as a catalyst for sensing and electroanalysis applications for their superior reactivity and chemical robustness [33, 88–90]. In contrast to their counterparts of oxides (1 × 10^−3^ S m^−1^) and sulfides (9.7 × 10^−2^–10^3^ S m^−1^) [49, 91], molybdenum nitrides possess improved electronic conductivity. Utilizing silica as the template (Figures 12(a) and 12(b)), Jiang et al. prepared highly conductive mesoporous Mo_2_N (1 × 10^5^ S m^−1^, 121 m^2^ g^−1^) with an average pore size of 8.6 nm [92]. When mesoporous Mo_2_N were immersed into Li_2_S_6_ solution, the yellow color disappeared, suggesting its strong adsorption. Benefiting from these merits, the mesoporous Mo_2_N/S cathode showed high capacity retention of 92.0% (corresponding to 914 mAh g^−1^) after charged/discharged at 0.5 C for 100 cycles, better than that based on nonporous Mo_2_N. Similarly, Wang et al. synthesized the MoN@N-doped carbon (MoN-NC) porous octahedron using MOF ([Cu_2_(BTC)_4/3_(H_2_O)_2_]_6_[H_3_PMo_12_O_40_]; BTC (benzene-1,3,5-tricarboxylate)) as precursors followed by thermal annealing, etching, and nitridation at elevated temperature (Figure 12(c)) [93]. The CV results tested in a symmetric cell using the Li_2_S_6_-containing electrolyte indicated MoN-NC promoted the chemisorption and conversion of LiPSs. The MoN-NC/S cathode with 77.0 wt% sulfur loading had 88.0% capacity retention at 0.5 C with 934 mAh g^−1^ left after 100 cycles, superior to MoN/S (71.0%) and NC/S (49.0%).

The design of heterostructures is another viable way to enhance the performance of molybdenum nitrides. Ye et al. prepared the 2D MoN-VN nanosheets (~7.1 nm thick) with a lateral size of a few microns via a salt template method and employed them as the sulfur host to regulate LiPSs (Figures 12(d)–12(g)) [94]. The introduction of V atoms can tailor the electronic states of Mo sites on the MoN surface and enabled higher adsorption ability for V-MoN than MoN. The MoN-VN/S cathode demonstrated capacity retention of 72.0% with 555 mAh g^−1^ left after cycling at 1 C for 500 times.

**Figure 13:**
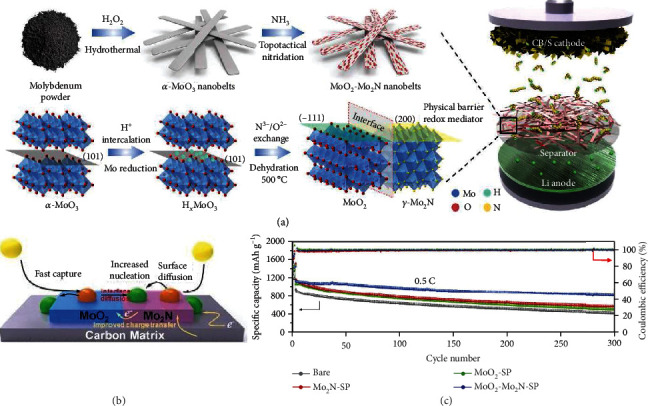
(a) Schematic illustration of the synthesis of the MoO_2_-Mo_2_N heterostructure. (b) The conversion of LiPSs and the nucleation of Li_2_S on the MoO_2_-Mo_2_N surface. (c) Cycling performance of the cells based on different separators. (a–c) Reproduced with permission from Elsevier [95].

Yang et al. developed an in situ topotactical nitridation strategy to prepare MoO_2_-Mo_2_N nanobelts that were incorporated as interlayer materials between the cathode and the separator in Li-S batteries (Figure 13) [95]. DFT calculation disclosed that the binding strength of MoO_2_ surfaces to Li_2_S_4_ is higher than that of Mo_2_N. The potentiostatic discharge tests of Li-Li_2_S_8_ batteries based on MoO_2_-Mo_2_N at 2.08 V exhibited a capacity of ~202 mAh g^−1^ for Li_2_S precipitation, better than that based on MoO_2_ (~103 mAh g^−1^) and Mo_2_N (~118 mAh g^−1^), confirming the accelerated conversion of LiPSs. Such heterostructures retained 73.6% (823 mAh g^−1^) after 300 cycles at 0.5 C. In another work, Li et al. proposed heterostructural MoO_2_-Mo_3_N_2_ holey nanobelts which exhibited improved electrochemical kinetics compared with their single-component counterparts (MoO_2_ or Mo_3_N_2_) [96]. This noncarbon heterojunction substrate enabled a high loading level of 75.0 wt% sulfur. The initial capacity of MoO_2_-Mo_3_N_2_/S with 75.0 wt% of sulfur loading retained 762 mAh g^−1^ (corresponding to 76.0% of initial capacity) after cycling at 0.5 C for 1000 times. Alternatively, Chen et al. coated molybdenum nitride nanosheets, which were obtained through a salt template method, on the Celgard separator (denoted as MoN*_x_*/Celgard), and the as-assembled Li-S batteries delivered a capacity of 566 mAh g^−1^ after 500 cycles at 0.5 C, corresponding to 68.1% retention [97].

**Figure 14:**
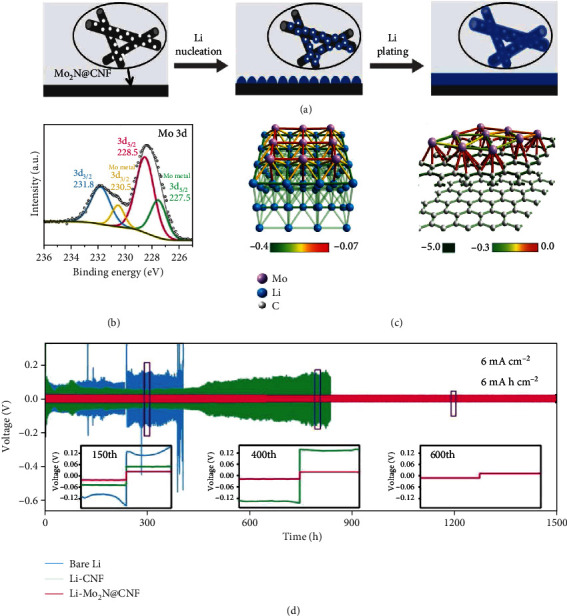
(a) The nucleation and subsequent plating process on Mo_2_N@CNF. (b) XPS spectrum of Mo 3d in lithiated Mo_2_N@CNF after bombarded by Ar plasma for 5 min. (c) Theoretical calculation of the interatomic interaction between Mo-Li (left) and C-Li (right) by using the crystal orbital Hamilton population (COHP). (d) Cycling tests of the symmetric cells composed of bare Li, Li-CNF, and Li-Mo_2_N@CNF anodes at 6 mA cm^−2^. (a–c) Reproduced with permission from Wiley-VCH [98].

Very recently, the Mo_2_N@CNF matrix was prepared by thermally annealing the hybrid film of CNF and (NH_4_)_6_Mo_7_O_24_∙4H_2_O at 800°C and then used as a scaffold for homogenous Li plating (Figure 14(a)) [98]. The XPS spectrum of Mo 3d in lithiated Mo_2_N@CNF after etched by Ar plasma unveiled that Mo_2_N reacted with Li generating Mo metal and Li_3_N via 3Li + Mo_2_N → 2Mo + Li_3_N (Figure 14(b)). In addition to the matchable lattice between Li and Mo, a theoretical calculation based on crystal orbital Hamilton population (COHP) indicated that Li tends to bond with Mo rather than Li due to the higher strength (Figure 14(c)), resulting in the uniform nucleation and subsequent deposition of Li. The incorporation of Mo_2_N@CNF enabled the symmetric cell outstanding cycling stability at 6 mA cm^−2^ for 1500 h (Figure 14(d)).

Molybdenum nitrides have excellent electrical conductivity, high catalytic properties, and robust structure, which are beneficial for accelerating the conversion of LiPSs and alleviating electrode fragmentation caused by the volume expansion of the sulfur cathodes. Furthermore, their unique lithophilicity can guide the homogeneous electrodeposition of Li metal, thereby alleviating the dendritic growth. However, similar to molybdenum oxides, molybdenum nitrides generally have low specific surface areas and lack a facile synthetic strategy [33].

## 5. Molybdenum Carbides

**Figure 15:**
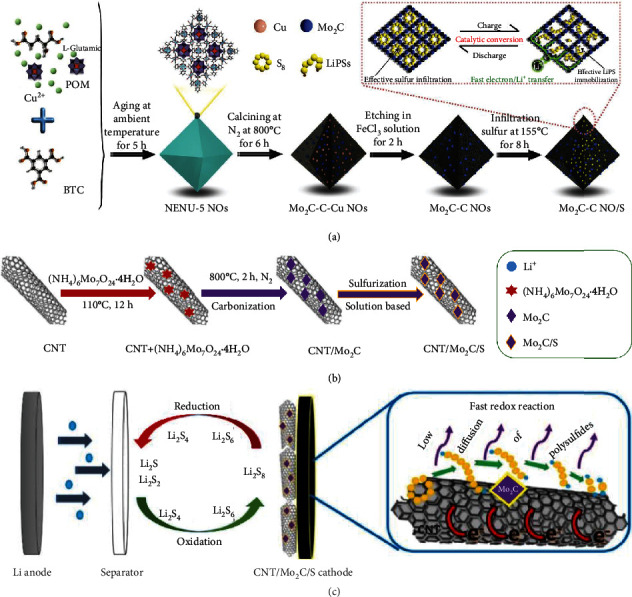
(a) The preparation of the MoC_2_-C NO/S cathode. Reproduced with permission from Elsevier [109]. (b) Schematic illustration of the synthesis of CNT/Mo_2_C and CNT/Mo_2_C/S. (c) Fast conversions of LiPSs on CNT/Mo_2_C via adsorption and subsequent redox reaction. (b, c) Reproduced with permission from IOP Publishing [120].

Owing to its high catalytic activity, low cost, and good conductivity, molybdenum carbide has been widely studied during the past decades for catalysis [33, 121]. Chen et al. synthesized porous Mo_2_C-C with a surface area of 196 m^2^ g^−1^ through pyrolyzing Mo-based MOF at 800°C followed by FeCl_3_ etching (Figure 15(a)) [109]. The hybrid showed effective adsorption to LiPSs while the ultrafine *β*-Mo_2_C nanocrystals encapsulated in carbon accelerated the redox kinetics toward LiPS conversion. As a result, the Mo_2_C-C NO/S cathode containing ~1.1 mg cm^−2^ sulfur delivered a capacity of 762 mAh g^−1^ (72.5% of the initial value) after the cycling test at 1 C for 600 times. Razaq et al. anchored Mo_2_C nanoparticles on carbon nanotubes (CNT/Mo_2_C) by annealing the mixture of CNTs and ammonium molybdate at 800°C (Figures 15(b) and 15(c)) [120]. The strong binding between Mo_2_C and CNTs ensured a highly conductive pathway for efficient electron transfer, and the porous assembly guaranteed rapid electrolyte infiltration, while the combination (CNT/Mo_2_C) prompted redox reactions of LiPSs. At 2 C, CNT/Mo_2_C/S exhibited 417 mAh g^−1^ (corresponding to capacity retention of 52.0%) after cycling 900 times. Similarly, Shang et al. achieved a device capacity of 1086 mAh g^−1^ at 0.2 C by decorating Mo_2_C nanoparticles on N-doped carbon nanofibers (Mo_2_C-NCNF) as the sulfur host for Li-S batteries [107]. Li et al. embedded necklace-like MoC in N-doped carbon nanofibers (MoC@N-CNF) using bacterial cellulose as a carbon source. The as-fabricated MoC@N-CNF/S cathode containing 10.0 mg cm^−2^ sulfur provided a capacity of 911 mAh g^−1^ at 1 C retaining 70.6% after 350 cycles.

**Figure 16:**
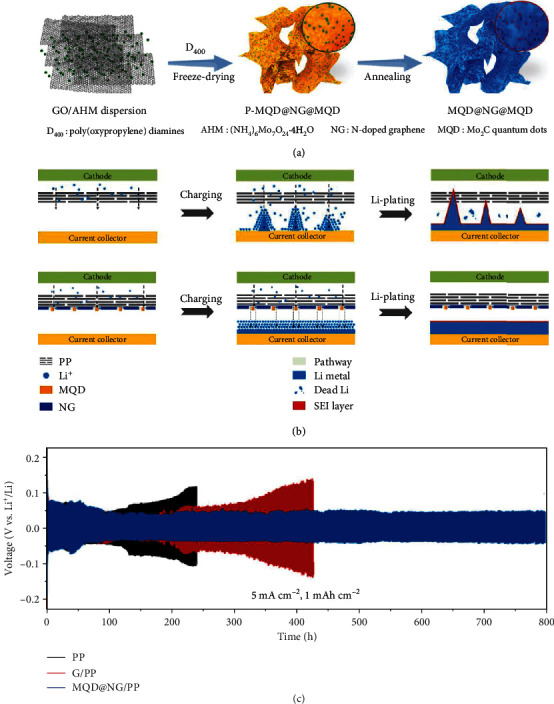
(a) The loading of Mo_2_C quantum dots (MQDs) on N-doped graphene (NG) under the assistance of poly(oxypropylene) diamines (D_400_). (b) Li plating process in Li-S batteries, respectively, using PP and MOD@NG/PP separators. (c) Voltage-time profiles of symmetric cells, respectively, using PP, G/PP, and MOD@NG/PP separators at 5 mA cm^−2^ (1 mAh cm^−2^). Reproduced with permission from Elsevier [113].

Apart from the application as the cathode host in suppressing the shuttle effect, the lithiophilic Mo_2_C also has the ability to facilitate the uniform Li deposition on anodes. In a recent work, an interlayer between the separator and the anode was prepared by uniformly anchoring Mo_2_C quantum dots (MQDs) on N-doped graphene (MQD@NG) under the assistance of poly(oxypropylene) diamines (D_400_) (Figure 16(a)) [113]. The experimental results demonstrated that the presence of the MQD@NG interlayer effectively suppressed the Li dendrites. In contrast to the PP separator, the lithiophilic MQD@NG-modified PP separator possessing fast ion diffusion pathways promoted uniform Li^+^ flow to the surface of Li metal anodes, leading to homogeneous dendrite-free Li deposition. As a result, the Li|Li symmetric cell with the MQD@NG/PP separator showed stable voltage-time profiles with small hysteresis over 800 h at 5 mA cm^−2^ and 1 mAh cm^−2^, better than that composed of the bare PP separator (130 h) and G/PP (~200 h).

Molybdenum carbides have similar properties to molybdenum nitrides with excellent metallic conductivity, high catalytic activity for LiPS conversion, good affinity to Li for uniform plating, yet generally low specific surface areas [33]. In addition, The preparation of molybdenum carbides typically involves high-temperature calcination under a reductive or inert atmosphere, making the process costly and complicated.

## 6. Molybdenum Phosphides

**Figure 17:**
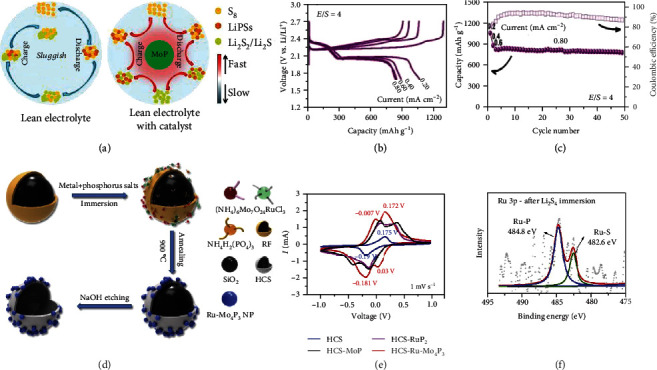
(a) Schematic illustrations of possible reaction pathways for sulfur cathodes with and without the MoP catalyst under lean electrolyte condition. (b) Representative charge/discharge curves at various rates and (c) stability test of the MoP-CNT/S electrode under the *E*/*S* = 4 condition. (a–c) Reproduced with permission from Wiley-VCH [126]. (d) The preparation of HCS-Ru-Mo_4_P_3_ NPs. (e) CV profiles of the asymmetric cells based on HCS, HCS-MoP, HCS-RuP_2_, and HCS-Ru-Mo_4_P_3_ hosts with 0.1 M Li_2_S_8_ in the catholyte. (f) The XPS spectra of Ru 3p in HCS-Ru-Mo_4_P_3_ after Li_2_S_4_ immersion. (d–f) Reproduced with permission from Elsevier [127].

Transition metal phosphides (TMPs) are a kind of widely utilized active materials in catalysis and energy storage for their high conductivity and stability [122, 123]. Particularly, molybdenum phosphide is a well-known catalyst for the hydrodesulfurization process in the petroleum industry because of its chemical interaction with sulfur species [124, 125]. Inspirited by this principle, Yang et al. synthesized the MoP-CNT hybrid by hydrolysis of (NH_4_)_6_Mo_7_O_24_ to MoO*_x_* followed by phosphorization in PH_3_ and verified the electrocatalytic properties of MoP nanoparticles (Figures 17(a)–17(c)) [126]. The MoP-CNT/S cathode containing 10.0 wt% of MoP-CNT showed a capacity of ~830 mAh g^−1^ without any obvious decay over 50 cycles at 0.8 mA cm^−2^.

Phase engineering and heteroatom doping are considered two effective strategies to adjust the properties of the catalyst [128, 129]. Following this idea, Ma et al. transformed MoP to Mo_4_P_3_ via Ru doping (Ru-Mo_4_P_3_) and demonstrated that Ru-Mo_4_P_3_ can effectively facilitate the electrocatalytic conversion of LiPSs (Figures 17(c)–17(f)) [127]. The separation between the cathodic and anodic peaks was ~0.18 V for the devices composed of HCS-Ru-Mo_4_P_3_, suggesting an accelerated LiPS conversion. The enhanced catalytic activity was attributed to two aspects: (i) compared to MoP, the Mo/P ratio in Mo_4_P_3_ became higher, exposing more Mo sites, and (ii) the Ru doping optimized the adsorption/desorption of reaction intermediates on Mo sites [130–132]. The HCS-Ru-Mo_4_P_3_/S cathode delivered 1178 mAh g^−1^ and 660 mAh g^−1^ at 0.5 C and 4 C in the Li-S battery, respectively.

**Figure 18:**
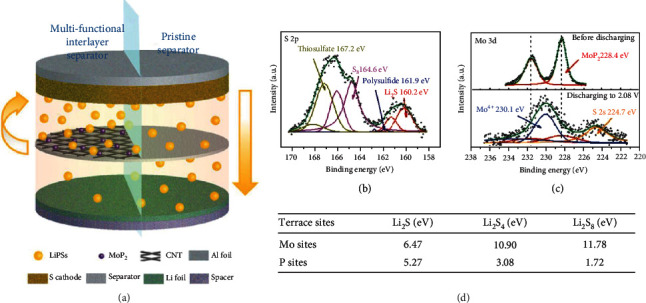
(a) Li-S cells with (left) and without (right) the modification of MoP_2_/CNT on commercial separators. XPS spectra of (b) S 2p and (c) Mo 3d in CNT/MoP_2_ before and after cycling. (d) The calculated binding energies between MoP_2_ and different LiPSs. (a–d) Reproduced with permission from Wiley-VCH [116].

By drop casting molybdenum diphosphide (MoP_2_) nanoparticles on superaligned CNT films that were cross-stacked together, Luo et al. designed a multifunctional interlayer on the Celgard 2400 separator (Figure 18) [116]. According to X-ray photoelectron spectroscopy (XPS) characterization, when the battery was discharged to 2.08 V, Mo^4+^ in the Mo 3d spectrum was detected, suggesting that the oxidation of Li_2_S_4_ to thiosulfate may be accomplished by Mo^6+^. Li_2_S_4_ and Li_2_S_2_ Raman peaks were only observed on the side of the CNT/MoP_2_ interlayer facing to the cathode, indicating that LiPSs were effectively blocked because of the physical hindrance of CNT films and the catalytic contribution of Mo sites. This was further confirmed by DFT calculations, which showed that high-order LiPSs (Li_2_S_4_ and Li_2_S_8_) had much larger binding energy to Mo sites in comparison with P sites. The CNT/MoP_2_ modification enabled the as-fabricated Li-S battery 1223 mAh g^−1^ discharging capacity at 0.2 C with retention of 74.0% after 100 cycles.

Molybdenum phosphides comparably exhibit superior catalytic performance for the catalytic conversion of LiPSs even under lean electrolyte conditions, which is beneficial to increase the energy density of Li-S batteries. The Mo centers are believed to be the active sites for the adsorption and electrocatalytic conversion of LiPSs. Although molybdenum phosphides can be synthesized under a relatively mild condition, compared with molybdenum carbides and molybdenum nitrides, using NH_4_H_2_PO_4_ and NaH_2_PO_2_ as the P sources, toxic gas (e.g., PH_3_) is generated during phosphorization and phosphates are inclined to be oxidized in air.

## 7. Molybdenum Metal

**Figure 19:**
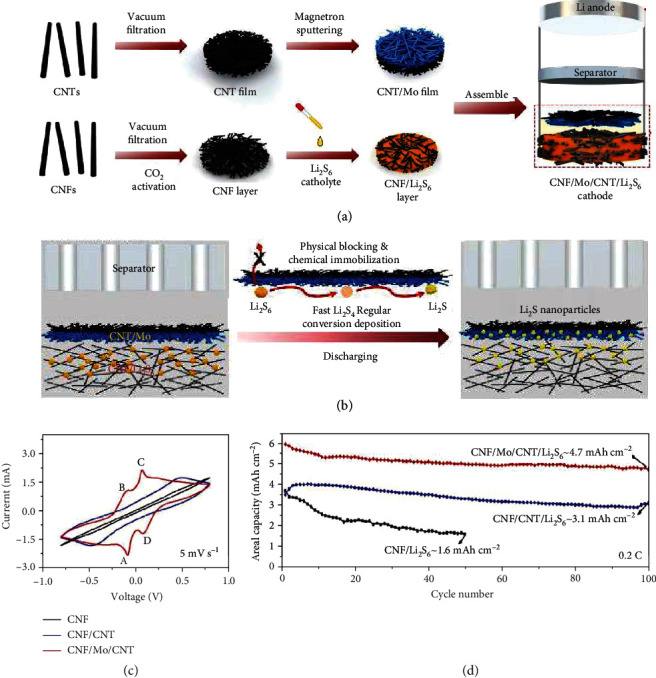
(a) The assembly of the Li-S battery based on the CNF/Mo/CNT/Li_2_S_6_ cathode. (b) The electrochemical behavior of the CNF/Mo/CNT/Li_2_S_6_ cathode with the Li_2_S_6_ catholyte. (c) CV curves of the symmetric cells employing CNF/Mo/CNT, CNF/CNT, and CNF electrodes at 5 mV s^−1^. (d) Cycling performances of Li-S batteries based on CNF/Mo/CNT/Li_2_S_6_, CNF/CNT/Li_2_S_6_, and CNF/Li_2_S_6_ electrodes under a high sulfur mass loading (7.6 mg cm^−2^). (a–d) Reproduced with permission from the American Chemical Society [118].

Very recently, Li et al. prepared a Mo/CNT thin film by a magnetron sputtering technique and used it as an interlayer in Li-S batteries (Figure 19) [118]. It was claimed that the sulfur-passivated Mo nanoclusters (~0.05 mg cm^−2^) in Mo/CNTs acted as capturing sites and catalytic centers for the chemical immobilization and conversion of LiPSs, while the compact CNT film functioned as a physical blocker for inhibiting the LiPS shuttle. As a result, the battery self-discharge was effectively suppressed and 722 mAh g^−1^ was achieved at 1 C with 65.0% retained after 500 cycles.

To date, there are only a few works reporting the direct utilization of Mo metal in Li-S batteries. According to these works of literature, the adsorption and catalytic properties of Mo metal are attributed to the formation of Mo-S bonds, yet further pieces of evidence are required. In addition, the binding energy theoretically follows the sequence of Mo-Mo>Mo-Li>Li-Li [98]. Therefore, the uniform loading of Mo nanoparticles on a high specific surface substrate may have the potential for the protection of Li metal anodes.

## 8. Conclusions and Prospects

We have comprehensively summarized the recent progress on Mo-based materials for Li-S batteries. Comparably, molybdenum oxides show strong adsorption capability toward LiPSs due to their polar Mo-O bond. However, the reaction kinetics of absorbed LiPSs are lowered by their poor intrinsic conductivity. Comparably, molybdenum dichalcogenides have improved conductivity, moderate binding energy, and catalytic performance with active centers mainly concentrating at the edge and defect sites. Molybdenum nitrides, carbides, and phosphides possess high electronic conductivity, excellent catalytic properties, and chemical durability in the organic electrolyte (without reacting with Li), which are promising materials for capturing LiPSs and catalyzing their redox reaction. Moreover, these materials as a scaffold facilitate the uniform deposition of insoluble Li_2_S, thus alleviating the shuttle effect. Furthermore, the unique lithophilicity of molybdenum nitrides and carbides facilitates the uniform electroplating of Li metal, thereby alleviating the dendritic growth of Li metal anodes. Although their catalytic performance can be further enhanced by reducing particle sizes so as to expose more electrochemical active surfaces, complicated procedures and harmful gases (e.g., NH_3_ and PH_3_) are inevitably involved. Mo metal shows the highest electronic conductivity and moderate catalytic activity (probably due to the formation of Mo-S bonds) toward the conversion of LiPSs. However, Mo metal can be oxidized by O_2_ in air and react with sulfur species during the charge/discharge process, forming MoO*_x_* and Mo-S bonds on its surface, thereby hindering the electron transport during the reaction [39].

In addition to the abovementioned analysis, there are several issues that need future endeavors:

1. Although many materials have demonstrated catalytic capability on the conversion of LiPSs [133], there is currently a lack of criteria to horizontally evaluate and compare their catalytic performance. Therefore, it is of great importance to take the physicochemical properties of Mo-based materials into consideration. For example, the catalytic capability of MoS_2_ is related to their different crystal structures, types of defects, and/or active sites (or facets) exposed. Moreover, the redox potential of sulfur hosts versus lithium was reported to be the key parameter for the adsorption and subsequent conversion of LiPSs [31]. As a result, it is highly necessary to exploit advance in situ/ex situ techniques to identify the role of high-valence Mo atoms during the catalysis
2. An in-depth understanding of the chemical scission of the S-S bond is necessitated. The conversion of LiPSs accompanied by a series of chemical processes severely depends on the chemical state of the material surface. The coordination state of Mo atoms on the surface of Mo-based materials has a significant impact on the adsorption and catalysis of LiPSs. For instance, Sun et al. revealed that Mo_2_C (101) surfaces underwent a sulfurization process during the sulfur loading and the resultant sulfurized Mo_2_C showed a similar mechanism of adsorption and catalytic activity to that of TMDs [134]
3. Material design is believed to be an effective strategy for promoting the performance of Mo-based materials in Li-S batteries (Table 1). For example, the Mo-based materials are expected to have high electronic conductivity, strong affinity to LiPSs (or Li^+^), excellent catalytic capability, large specific surface areas, and uniform loading (dispersion) to ensure full utilization of cathode sulfur, efficient capture of LiPSs and subsequent conversion, high energy density, and dendrite-free Li plating. Several strategies that are frequently adopted for improving the performance of Mo-based materials include the introduction of defection/heteroatoms (enriching active sites and enhancing conductivity), the hybridization with conductive carbonaceous materials (e.g., rGO, CNT, and CNF for improving conductivity), the synthesis of hierarchical structures (enlarging active sites and providing physical blockage), and the design of heterostructures (engineering the adsorption and catalytic properties)
4. It is highly desired to develop a scalable, cost-effective, and environmentally friendly method for synthesizing cathode materials toward the commercialization of Li-S batteries. The state-of-the-art strategies reported in the lab typically involve complicated procedures, expensive equipment, toxic substances (e.g., gases and solvents), and high-temperature calcination, which are unfavorable for mass production. In addition, the low sulfur loading, typically 0.5–2.0 mg cm^−2^ as reported in the literature, further hinders the practical application of Li-S batteries with the target energy density of ~500 W h kg^−1^

## Figures and Tables

**Table 1:** The applications of Mo-based materials in Li-S batteries.

Classification		Material	Preparation method (design strategies)	Structure characteristics	Sulfur content (wt%), areal sulfur loading (mg cm^−2^), areal loading on the separator (mg cm^−2^)	Electrolyte dosage (*μ*L mg^−1^)	Retained capacity (mAh g^−1^), capacity retention (%), cycle number, and rate (C)	Voltage range (V)	Reference
Molybdenum oxides	Cathode	MoO_3_/CNT/S	Alternative filtration (hybridizing with carbonaceous materials)	Double-layer membrane	28.8 wt%, 1.0 mg cm^−2^, N/A	N/A	666 mAh g^−1^, 62.0%, 350, 0.5 C	1.8–2.8 V	[52]
		MoO_3−*x*_/S	Hydrothermal reaction and annealing (introducing oxygen defects)	Nanobelt	47.0 wt%, 1.0 mg cm^−2^, N/A	N/A	480 mAh g^−1^, 62.0%, 600, 1 C	1.7–2.6 V	[47]
		Sn_0.063_MoO_3_/S	Solvothermal reaction (intercalating heteroatoms)	Nanoribbon	67.4 wt%, ~1.3 mg cm^−2^, N/A	N/A	721 mAh g^−1^, 80.0%, 500, 1 C	1.8–2.8 V	[48]
		MoO_3_-CP/S	Hydrothermal reaction (hybridizing with carbonaceous materials)	Nanoflake	N/A, 3.0 mg cm^−2^, N/A	N/A	976 mAh g^−1^, 99.5%, 1000, 1 C	1.7–2.8 V	[99]
		MoO_2_/C-NC/S	Calcination and etching (constructing nanostructure by MOF template)	Porous framework	76.3 wt%, 1.1 mg cm^−2^, N/A	N/A	400 mAh g^−1^, 52.7%, 1000, 2 C	1.7–2.8 V	[60]
		MoO_2_/G/S	Hydrothermal reaction and annealing (constructing nanostructure)	Hollow sphere	79.0 wt%, N/A, N/A	N/A	905 mAh g^−1^, 80.5%, 100, 0.2 C	1.7–2.8 V	[59]
		MoO_2_/S	Calcination and etching (constructing nanostructure by silicon template)	Mesoporous framework	38.0 wt%, 1.0 mg cm^−2^, N/A	N/A	570 mAh g^−1^, 51.8%, 250, 0.1 C	1.7–2.8 V	[100]
	Separator	MoO_3_@CNT	Hydrothermal reaction and filtration (hybridizing with carbonaceous materials)	Scaffold-like network	N/A, 0.6 mg cm^−2^, ~0.6 mg cm^−2^	N/A	755 mAh g^−1^, 53.0%, 200, 0.3 C	1.5–3.5 V	[54]
		MoO_3_ separator	Hydrothermal reaction and slurry coating (constructing nanostructure)	Porous nanobelt layer	N/A, 1.0 mg cm^−2^, ~0.5 mg cm^−2^	N/A	684 mAh g^−1^, 49.0%, 200, 0.5 C	1.6–2.8 V	[53]
		MoO_2_-CNF	Electrospinning and calcination (hybridizing with carbonaceous materials)	Mesoporous nanofiber membrane	70.0 wt%, 2.5 mg cm^−2^, N/A	38 *μ*L mg^−1^	1006 mAh g^−1^, 73.6%, 150, 0.1 C	1.7–2.8 V	[63]
		MoO_3_/CNF	Electrospinning and calcination (hybridizing with carbonaceous materials)	Mesoporous nanofiber membrane	N/A, N/A, N/A	N/A	776 mAh g^−1^, 55.0%, 500, 0.5 C	1.7–2.7 V	[55]

Molybdenum sulfide	Cathode	MoS_2−*x*_/rGO/S	Filtration and calcination (introducing sulfur defects)	Nanoflake	75.0 wt%, 0.9 mg cm^−2^, N/A	55 *μ*L mg^−1^	820 mAh g^−1^, 70.6%, 150, 0.5 C	1.8–2.6 V	[71]
		P-Mo_0.9_Co_0.1_S_2_/S	Hydrothermal reaction (introducing heteroatoms)	Core-shell nanotube	N/A, 2.0 mg cm^−2^, N/A	N/A	1187 mAh g^−1^, 89.0%, 150, 0.5 C	1.7–2.6 V	[72]
		GA-DR-MoS_2_/S	Hydrothermal reaction and freeze-drying (constructing nanostructure by ice template)	Porous 3D aerogel	70.0 wt%, N/A, N/A	N/A	821 mAh g^−1^, 57.8%, 500, 0.2 C	1.7–2.8 V	[78]
		rGO-MoS_2_ QD/S	Hydrothermal reaction (constructing nanostructure)	Quantum dot	50.0 wt%, ~1.4 mg cm^−2^, N/A	15 *μ*L mg^−1^	503 mAh g^−1^, 99.3%, 300, 2 C	1.8–2.8 V	[101]
		MoS_2_/S	Intercalation exfoliation and electrostatic assembly (constructing nanostructure)	Hollow sphere	65.0 wt%, 1.5 mg cm^−2^, N/A	N/A	585 mAh g^−1^, 44.9%, 1000, 1 C	1.8–2.6 V	[102]
		MoS_2_/CNT/S	Drop casting (hybridizing with carbonaceous materials)	Cross-stacked membrane	N/A, 2.6 mg cm^−2^, N/A	N/A	855 mAh g^−1^, 58.0%, 50, 0.2 C	1.5–2.8 V	[103]
		MoS_2_/g-C_3_N_4_/S	Recrystallization and calcination (hybridizing with polar materials)	Nanosheet	59.0 wt%, 1.5 mg cm^−2^, N/A	18 *μ*L mg^−1^	569 mAh g^−1^, 73.2%, 400, 1 C	1.8–2.8 V	[104]
	Separator	Edg-MoS_2_/C HMs	Hydrothermal reaction and calcination (constructing nanostructure)	Hollow sphere	64.0 wt%, 6.1 mg cm^−2^, 0.3 mg cm^−2^	12 *μ*L mg^−1^	478 mAh g^−1^, 86.3%, 300, 0.5 C	1.8–2.7 V	[81]
		MoS_2_/Celgard	Intercalation exfoliation and filtration (constructing nanostructure)	Nanosheet	65.0 wt%, N/A, N/A	N/A	401 mAh g^−1^, 49.6%, 600, 0.5 C	1.5–3.0 V	[79]
		(M-P/P)_10_	Intercalation exfoliation and electrostatic assembly (hybridizing with polar materials)	Nanosheet	60.0 wt%, 1.2 mg cm^−2^, 0.1 mg cm^−2^	N/A	423 mAh g^−1^, 42.0%, 2000, 1 C	1.7–2.6 V	[80]
	Anode	MoS_2_-coated Li metal	Sputtering (constructing protective layer)	Nanosheet	33.0 wt%, 3.4 mg cm^−2^, N/A	N/A	940 mAh g^−1^, 84.0%, 1200, 0.5 C	1.5–3.0 V	[82]

Molybdenum selenide	Cathode	MoSe_2_@rGO/S	Hydrothermal reaction (hybridizing with carbonaceous materials)	Nanoflake	75.8 wt%, 1.7 mg cm^−2^, N/A	8 *μ*L mg^−1^	1086 mAh g^−1^, 67.5%, 250, 0.25 C	1.65–2.8 V	[83]
		MoSe_2_/N-rGO/S	Freeze-drying and annealing (constructing nanostructure)	Nanoflake	62.0 wt%, 1.1 mg cm^−2^, N/A	N/A	887 mAh g^−1^, 86.3%, 100, 0.2 C	1.8–2.8 V	[84]

Molybdenum nitrides	Cathode	Mesoporous Mo_2_N/S	Annealing and etching (constructing nanostructure by silicon template)	Mesoporous framework	48.2 wt%, 1.1 mg cm^−2^, N/A	N/A	914 mAh g^−1^, 91.9%, 100, 0.5 C	1.7–2.8 V	[92]
		MoN-NC/S	Annealing and etching (constructing nanostructure by MOF template)	Porous framework	77.0 wt%, 1.5 mg cm^−2^, N/A	N/A	895 mAh g^−1^, 71.0%, 100, 0.5 C	1.7–2.8 V	[93]
		Mo_2_N/S	Annealing (constructing nanostructure)	Mesoporous nanorod	N/A, 8.0 mg cm^−2^, N/A	N/A	573 mAh g^−1^, 57.2%, 100, 0.1 C	1.5–3.0 V	[37]
	Separator	MoN*_x_*	Recrystallization and annealing (constructing nanostructure by salt template)	Nanosheet	73.0 wt%, ~1.4 mg cm^−2^, 0.4 mg cm^−2^	N/A	566 mAh g^−1^, 68.1%, 500, 0.5 C	1.7–2.6 V	[97]
		MoN-G/PP	Hydrothermal reaction and annealing (hybridizing with carbonaceous materials)	Nanosheet	90.0 wt%, 1.2 mg cm^−2^, N/A	N/A	678 mAh g^−1^, 63.9%, 500, 0.5 C	1.8–2.8 V	[105]

Molybdenum carbides	Cathode	*β*-Mo_2_C/CNF/S	Electrospinning and annealing (constructing nanostructure)	Porous nanofiber membrane	48.2 wt%, 1.5 mg cm^−2^, N/A	N/A	767 mAh g^−1^, 75.4%, 50, 0.1 C	1.7–2.8 V	[106]
		CNT/Mo_2_C/S	Electrospinning and annealing (constructing nanostructure)	Porous nanofiber membrane	70.0 wt%, 2.5 mg cm^−2^, N/A	N/A	718 mAh g^−1^, 86.0%, 100, 1 C	1.6–3.0 V	[107]
		Mo_2_C/C@C/S	Annealing (constructing nanostructure)	Hollow sphere	70.0 wt%, 1.0 mg cm^−2^, N/A	N/A	652 mAh g^−1^, 81.5%, 300, 1 C	1.7–2.8 V	[108]
		Mo_2_C-C NO/S	Annealing and etching (constructing nanostructure by MOF template)	Porous framework	72.2 wt%, 4.2 mg cm^−2^, N/A	30 *μ*L mg^−1^	623 mAh g^−1^, 77.2%, 100, 0.5 C	1.7–2.8 V	[109]
		Mo_2_C NP-CNF/S	Hydrothermal reaction and annealing (constructing nanostructure)	3D nanofiber network	N/A, ~1.8 mg cm^−2^, N/A	N/A	997 mAh g^−1^, 86.8%, 200, 0.2 C	1.8–2.6 V	[110]
		MoC_1−*x*_/C/S	Annealing (constructing nanostructure)	Nanoflower	N/A, ~0.8 mg cm^−2^, N/A	N/A	860 mAh g^−1^, 71.6%, 500, 0.48 C	1.8–2.6 V	[111]
		MoC@N-CNF/S	Freeze-drying and annealing (constructing nanostructure by ice template)	Nanofiber membrane	50.0 wt%, 1.5 mg cm^−2^, N/A	25 *μ*L mg^−1^	962 mAh g^−1^, 80.1%, 100, 0.3 C	1.7–2.8 V	[112]
	Separator	MQD@NG/PP	Freeze-drying and annealing (constructing nanostructure by ice template)	Quantum dots	80.0 wt%, 2.1 mg cm^−2^, N/A	20 *μ*L mg^−1^	1146 mAh g^−1^, 93.2%, 100, 0.2 C	1.7–2.8 V	[113]

Molybdenum phosphides	Cathode	MoP-CNT/S	Reflux and annealing (constructing nanostructure)	3D nanofiber network	72.0 wt%, 6.0 mg cm^−2^, N/A	5 *μ*L mg^−1^	830 mAh g^−1^, N/A, 50, 0.08 C	1.7–2.7 V	[114]
	Separator	MoP/rGO	Hydrothermal reaction and annealing (hybridizing with carbonaceous materials)	Nanosheet	77.0 wt%, 3.9 mg cm^−2^, 0.4 mg cm^−2^	N/A	760 mAh g^−1^, 86.4%, 300, 0.5 C	1.8–2.8 V	[115]
		MoP_2_/CNT	Reflux and drop casting (constructing nanostructure)	Nanofiber membrane	N/A, 1.2 mg cm^−2^, 0.3 mg cm^−2^	N/A	543 mAh g^−1^, 87.7%, 500, 0.1 C	1.8–2.6 V	[116]
		MoP@C/N HCSs	Annealing (constructing nanostructure)	Hollow sphere	75.0 wt%, 1.5 mg cm^−2^, 0.8 mg cm^−2^	N/A	1185 mAh g^−1^, 97.3%, 100, 0.2 C	1.7–2.8 V	[117]

Molybdenum metal	Cathode	Mo powder/S	Commercial product	Particle	60.0 wt%, ~1.1 mg cm^−2^, N/A	N/A	1108 mAh g^−1^, 95.0%, 130, 0.1 C	1.5–3.0 V	[39]
	Separator	CNT/Mo	Filtration and magnetron sputtering (hybridizing with carbonaceous materials)	Nanofiber membrane	N/A, 7.6 mg cm^−2^, 0.05 mg cm^−2^	N/A	621 mAh g^−1^, 80.0%, 100, 0.2 C	1.7–2.8 V	[118]

Heterojunctions	Cathode	C@SnO_2_/TMS/S	Hydrothermal reaction and calcination (hybridizing with polar materials)	Hierarchical nanosheet	N/A, ~2.8 mg cm^−2^, N/A	N/A	710 mAh g^−1^, 63.0%, 4000, 5 C	1.7–2.8 V	[70]
		2D MoN-VN/S	Recrystallization and annealing (hybridizing with polar materials by salt template)	2D sheet	58.5 wt%, 3.0 mg cm^−2^, N/A	N/A	555 mAh g^−1^, 72.0%, 500, 1 C	1.7–2.8 V	[94]
		MoO_2_-Mo_3_N_2_/S	Hydrothermal reaction and annealing (hybridizing with polar materials)	Porous nanobelt	75.0 wt%, 3.2 mg cm^−2^, N/A	N/A	451 mAh g^−1^, 69.0%, 1000, 0.5 C	1.8–3.0 V	[96]
	Separator	MoO_2_-Mo_2_N	Annealing (hybridizing with polar materials)	Nanobelt	73.0 wt%, 3.1 mg cm^−2^, 0.5 mg cm^−2^	7 *μ*L mg^−1^	790 mAh g^−1^, 74.0%, 100, 0.1 C	1.7–2.8 V	[95]
		MoP/MoS_2_@C	Annealing (hybridizing with polar materials)	Nanoparticle	70.0 wt%, 2.1 mg cm^−2^, N/A	15 *μ*L mg^−1^	650 mAh g^−1^, 59.0%, 500, 1 C	1.7–2.8 V	[119]
